# An Atypical HNF4A Mutation Which Does Not Conform to the Classic Presentation of HNF4A-MODY

**DOI:** 10.1155/2018/1560472

**Published:** 2018-05-28

**Authors:** Andrew J. Spiro, Katherine N. Vu, Alicia Lynn Warnock

**Affiliations:** ^1^Department of Internal Medicine, Walter Reed National Military Medical Center, Bethesda, MD, USA; ^2^Department of Endocrinology, Metabolism and Diabetes, Walter Reed National Military Medical Center, Bethesda, MD, USA; ^3^Uniformed Services University of Health Sciences, Bethesda, MD, USA

## Abstract

**Objective:**

To present the case of an atypical Hepatocyte Nuclear Factor 4 Alpha* (HNF4A)* mutation that is not consistent with the classically published presentation of* HNF4A*-Mature Onset Diabetes of the Young (MODY).

**Methods:**

Clinical presentation and literature review.

**Results:**

A 43-year-old nonobese man was referred to the endocrinology clinic for evaluation of elevated fasting blood glucose (FBG) measurements. Laboratory review revealed prediabetes and hypertriglyceridemia for the previous decade. Testing of autoantibodies for type 1 diabetes was negative. Genetic testing showed an autosomal dominant, heterozygous missense mutation (c.991C>T; p.Arg331Cys) in the* HNF4A* gene, which is correlated with* HNF4A*-MODY. Phenotypically, patients with an* HNF4A*-MODY tend to have early-onset diabetes, microvascular complications, low triglyceride levels, increased birth weight, fetal macrosomia, and less commonly neonatal hyperinsulinemic hypoglycemia. The patient did not demonstrate any of these features but instead presented with late-onset diabetes, an elevated triglyceride level, and a normal birth weight.

**Conclusion:**

Our patient likely represents an atypical variant of* HNF4A-*MODY with a milder clinical presentation. Patients with atypical, less-severe presentations of* HNF4A*-MODY may be largely undiagnosed or misdiagnosed, but identification is important due to implications for treatment, pregnancy, and screening of family members.

## 1. Introduction

MODY describes a cluster of monogenic disorders that are inherited in an autosomal dominant manner. The classic depiction of MODY is diabetes that presents before the age of 25 years, is not insulin-dependent, and shows no evidence of autoimmune pathology or insulin resistance. While MODY is only responsible for 1% of all cases of diabetes, it is the cause for 5% of people diagnosed before 45 years [1]. To date, several MODY-causing genes have been identified but the most common are glucokinase* (GCK)*,* HNF1A,* and* HNF4A* [1]. The* HNF4A*-MODY (MODY 1) and* HNF1A*-MODY (MODY 3) phenotypes resemble one another, producing significant pancreatic *β*-cell dysfunction which results in hyperglycemia and microvascular complications, whereas* GCK*-MODY (MODY 2) produces a mild hyperglycemia, which often does not require treatment [1] (see Table 1). A recent large-scale study in the United Kingdom showed that* HNF4A*-MODY (MODY 1),* GCK*-MODY (MODY 2), and* HNF1A*-MODY (MODY 3) represent 10%, 32%, and 52% of MODY cases in that population [2]. The prevalence of different MODY mutations varies from one country to another. In countries like France, Italy, and Spain, where asymptomatic glucose testing is common,* GCK*-MODY (MODY 2) is the most common subtype.* HNF1A*-MODY (MODY 3) is the most common subtype in countries where random glucose testing is less routine [3]. We describe a patient with a heterozygous missense mutation in* HNF4A* whose clinical presentation does not conform to the classically described features of the* HNF4A*-MODY.

## 2. Case Report

A 43-year-old nonobese man was referred to the endocrinology clinic for evaluation of elevated FBG measurements. His FBG and hemoglobin A1C (HbA1C) had been in the prediabetic range for the past decade, except for one elevated HbA1C of 6.7% (50 mmol/mol) two years prior (see Table 2). The patient denied any classic symptoms of diabetes. His medical history was notable for gout, hyperlipidemia, and hypertriglyceridemia. The patient was taking allopurinol and had been taking a moderate-intensity-statin for the previous 10 years. His mother had premature coronary artery disease (CAD) and type 2 diabetes (T2DM), controlled with oral medication. Physical exam showed a healthy, lean, and muscularly fit individual with a body mass index of 24.8 kg/m^2^. His latest HbA1C was 6.2% (44 mmol/mol) and recent FBG measurements were 131, 198, 115, and 188 mg/dL. An oral glucose tolerance test showed a result in the prediabetic range (178 mg/dL). Autoantibodies (Glutamate Decarboxylase 65 Ab, Pancreatic Islet Cell Ab, Islet Cells Ab 512, and Insulin Ab) were negative. His fasting insulin level was within normal limits while a C-peptide level was mildly elevated; however, these levels were checked at different time points and do not directly correlate with one another (see Table 2). Initially, his presentation was attributed to early well-controlled T2DM with elements of metabolic syndrome; however, due to his atypical physical appearance, he was referred for genetic testing for MODY. Testing revealed an autosomal dominant, heterozygous missense mutation (c.991C>T; p.Arg331Cys) in the* HNF4A* gene.

## 3. Discussion

As noted above,* HNF4A-*MODY (MODY 1) and* HNF1A-*MODY (MODY 3) are generally associated with the classic MODY features while* GCK-*MODY (MODY 2) has been recognized as a milder variant. The clinical manifestation of our patient's* HNF4A* mutation was atypical for multiple reasons. The primary atypical feature was that he did not meet laboratory criteria for diabetes diagnosis until the age of 41 (HbA1C = 6.7%; 50 mmol/mol), after which he was able to maintain his HbA1C levels in the prediabetic range with only lifestyle modification. Recent data showed that 71% of individuals with pathogenic* HNF4A* mutations developed diabetes by age of 30, with mean age of diagnosis in the early 20s. This data did not include the p.R114W mutation [4]. While uncommon, presentations later in life are not exceedingly rare. Another recent study reported approximately 82% penetrance of mutations at age of 40 years [5]. Aside from early-onset diabetes,* HNF4A* mutations have been associated with increased birth weight and macrosomia [6]. In one study, 56% of mutation carriers met criteria for macrosomia and carriers demonstrated a 790 g average increase in birth weight compared to noncarrier family members [7]. Our patient reported a birth weight between 6 and 7 lbs and had no history of macrosomia. Examination of the lipid profiles in* HNF4A*-MODY has revealed decreased serum triglycerides and Apo AII [6]. Evidence has also shown high LDL, low HDL, low apo CIII, and low apo B [1]. In contrast, our patient demonstrated consistent hypertriglyceridemia and normal-to-high levels of HDL and LDL. Apo B and Apo A-I were within the normal range.

The variance between our patient's clinical presentation and that of classic* HNF4A*-MODY can be explored by examining the function of the HNF4A protein. HNF4A is a transcription factor that is primarily expressed in the liver, in addition to the kidneys and pancreas [8]. It is not completely understood how* HNF4A* mutations result in impaired *β*-cell function and insulin secretion or alter the lipid profile. It is known though that HNF4A regulates expression of genes involved in glucose transport (GLUT2), glycolysis (aldolase B, liver pyruvate kinase), and lipid metabolism (Apo AII, apoB, and apoCIII) [8]. It has been proposed that low triglyceride concentrations found in typical* HNF4A*-MODY could result from the decreased transcriptional activity of the mutated HNF4A leading to less expression of apoB and apoCIII [8]. In our patient, the delayed and less-severe presentation of diabetes supports a milder impairment of *β*-cell function and insulin secretion in the pancreas. His elevated triglyceride level supports that expression of apoCIII and apoB has not been altered in the liver in the typical manner. It must be considered though that a definitive conclusion cannot be drawn based on a single patient. Other predisposing or protective factors should be weighed. Our patient's elevated triglyceride levels may not be related to his* HNF4A* mutation but instead due to a distinct and separate condition. With consideration of his mother's reported history of CAD and T2DM, he may have a separate predisposition to hypertriglyceridemia or metabolic syndrome.

There are more than 103* HNF4A*-MODY mutations in 173 families and there is great variation in the types of mutations (i.e., missense, frameshift, and nonsense) [6]. Thus, there is potential for a spectrum of phenotypes. Our patient likely represents a variant of* HNF4A-*MODY with a milder clinical presentation than the classic picture. A recent study which described the (p.R114W) mutation presented an* HNF4A*-MODY variant with a less-severe presentation of diabetes, noting a pathogenic penetrance of only 54% by age of 30 [4]. Patients with less-severe* HNF4A*-MODY variants, like those with the (p.R114W) mutation, present a particularly difficult population to diagnose and they may in fact be largely misdiagnosed. In the United Kingdom, it is estimated that >80% of MODY is misdiagnosed [2]. The milder degree of *β*-cell impairment and the delayed age of diagnosis can strongly mimic T2DM; however, clinical suspicion for MODY should increase in diabetics with negative autoimmune markers who do not demonstrate obesity and the metabolic syndrome [1]. Distinguishing* HNF4A*-MODY from T2DM has important implications for management. Sensitivity to sulfonylureas is well-established in* HNF4A*-MODY [6]. Use of sulfonylureas in* HNF4A*-MODY and* HNF1A*-MODY has been shown to improve glycemic control and can improve a patient's quality of life by avoiding insulin therapy [1]. Further,* HNF4A* mutations can have important ramifications during pregnancy. If either the mother or the father is known to be a mutation carrier, then there is increased risk of complications due to macrosomia, while the neonates who inherit the mutation are at risk of neonatal hypoglycemia [6]. Lastly, identifying an* HNF4A* mutation allows family members to be screened for carrier status and receive appropriate counselling. Genetic screening is recommended for all diabetic family members and genetic counselling is recommended for all nondiabetic members [1].

## 4. Conclusion

Our patient exhibited an atypical clinical presentation of an* HNF4A *mutation which likely represents a less-severe variant of* HNF4A*-MODY. Features of this milder variant include late-onset diabetes, hypertriglyceridemia, and a normal birth weight. There is great variation in the types of* HNF4A* mutations and potential for a spectrum of resultant phenotypes. Diagnosis of individuals who present with atypical, less-severe presentations of* HNF4A*-MODY is difficult, and they may be largely misdiagnosed. Identification of these patients is important due to implications for treatment, pregnancy, and the screening of family members.

## Figures and Tables

**Table 1:** Comparison of patient phenotype with the common MODY subtypes.

	Patient (*HNF4A* mutation)	*HNF4A*-MODY (MODY 1)	*HNF1A*-MODY (MODY 3)	*GCK*-MODY (MODY 2)
Frequency^*∗*^	–	≤10%	~30–50%	~30–50%

Age of Diagnosis	41 y/o	<25 y/o	<25 y/o	Usually age when blood glucose levels first measured

Pathophysiology	–	Transcription factor, decreased insulin secretion	Transcription factor, Decreased insulin secretion	Decreased glucose sensitivity, decreased glycogen storage

Characteristics	No current evidence of microvascular complications Managed with lifestyle modifications Hypertriglyceridemia, mildly elevated LDL and VLDL Normal Apo-B, Apo-A1	Microvascular complications Sensitive to sulfonylureas Low TG, high LDL, low HDL Low Apo A-II, C-III, B Fetal Macrosomia Neonatal hypoglycemia	Microvascular complications Sensitive to sulfonylureas Normal or high HDL Glycosuria	No microvascular complications Mild, generally does not require treatment

^*∗*^Percentage of occurrence among MODY patient with a genetic diagnosis. MODY = Mature Onset Diabetes of the Young; HNF = Hepatocyte Nuclear Factor; GCK = glucokinase; TG = triglyceride; LDL = low density lipoprotein; HDL = high density lipoprotein; VLDL = very low density lipoprotein; Apo = apolipoprotein. See reference [1].

**Table 2:** Patient laboratory data over time.

Age (years)	36.96	37.01	41.24	41.43	43.20	43.27	43.29	43.35
HbA1C (%)		5.7	6.7	6.3		6.2		
FBG (mg/dL)	123	113			131	198	115	188
C-peptide (ng/mL)								7.0
Fasting Insulin (mcU/mL)							14.7	
Glucose, 2 hr Post 75 gm Oral Glucose Load							178	
Total Cholesterol (mg/dL)	213	250	215	154	153			
HDL	52	47	37	39	38			
TG	277	342	397	165	144			
LDL	106	142	107	81	88			
BMI (kg/m^2^)		25.1	25.6	24.4		24.8		

HbA1C = hemoglobin A1C; FBG = fasting blood glucose; HDL = high density lipoprotein cholesterol; TG = triglyceride; LDL = low density lipoprotein cholesterol; BMI = body mass index; normal reference ranges for laboratory: (1) insulin (mcU/mL): (2.6–24.9); (2) C-peptide (ng/mL): (1.1–4.4). *Note*. For the duration listed, the patient was taking a moderate-intensity statin and not taking any medication for treatment of diabetes.

## Data Availability

The patients laboratory results are recorded and available in the military health care record system.
